# Prolactin receptor antagonism reduces the clonogenic capacity of breast cancer cells and potentiates doxorubicin and paclitaxel cytotoxicity

**DOI:** 10.1186/bcr2129

**Published:** 2008-08-05

**Authors:** Sacha J Howell, Elizabeth Anderson, Tom Hunter, Gillian Farnie, Robert B Clarke

**Affiliations:** 1 Breast Biology Group, Paterson Institute for Cancer Research, University of Manchester, Wilmslow Road, Manchester M20 4BX, UK; 2 Current address: Astra Zeneca UK, Alderley Park, Macclesfield SK10 4TG, UK

## Abstract

**Introduction:**

Exogenous prolactin is mitogenic and antiapoptotic in breast cancer cells, and overexpression of autocrine prolactin cDNA in breast cancer cell lines has been shown to stimulate their growth and to protect against chemotherapy-induced apoptosis. We examined the effects of the 'pure' prolactin receptor antagonist Δ1–9-G129R-hPrl (Δ1–9) on the breast cancer cell number and clonogenicity, alone and in combination with chemotherapy.

**Methods:**

The effects of doxorubicin, paclitaxel and Δ1–9 on the growth of breast cancer cell lines (MCF-7, T47D, MDA-MB-453, MDA-MB-468 and SK-BR-3) in monolayer culture were assessed by the sulphorhodamine B assay. Effects on clonogenicity were assessed by soft agar assay for the cell lines and by the mammosphere assay for disaggregated primary ductal carcinoma *in situ *samples. Dual-fluorescence immunocytochemistry was used to identify subpopulations of cells expressing the prolactin receptor and autocrine prolactin.

**Results:**

Δ1–9 as a single agent had no effect on the cell number in monolayer culture, but potentiated the cytotoxic effects of doxorubicin and paclitaxel. Doxorubicin accordingly induced expression of prolactin mRNA and protein in all five breast cancer cell lines tested. Δ1–9 alone inhibited the clonogenicity in soft agar of cell lines by ~90% and the mammosphere forming efficiency of six disaggregated primary ductal carcinoma *in situ *samples by a median of 56% (range 32% to 88%). Subpopulations of cells could be identified in the cell lines based on the prolactin receptor and prolactin expression.

**Conclusion:**

Autocrine prolactin appears to act as an inducible survival factor in a clonogenic subpopulation of breast cancer cells. The rational combination of cytotoxics and Δ1–9 may therefore improve outcomes in breast cancer therapy by targeting this cell population.

## Introduction

Exogenous prolactin has been shown to induce the proliferation, survival, migration and invasion of breast cancer cell lines *in vitro *and to increase the clonogenicity of primary human breast cancer samples in soft agar [1-5]. A prolactin excess reduces the tumour latency and increases the tumour incidence and growth rate in multiple rodent models of spontaneous and carcinogen-induced mammary tumours [6-8]. In humans, prospective case–control studies show that women with high versus low blood prolactin levels have an increased risk of preinvasive and invasive breast cancer [9,10]. Furthermore, the majority of human breast cancers have been shown to express the prolactin receptor (PRLR) [11].

Despite these observations, attempts to treat advanced breast cancer through the pharmacological inhibition of pituitary prolactin secretion, either as monotherapy or in combination with tamoxifen, have been disappointing. In a recent study of women with metastatic breast cancer, however, a significant increase in the objective tumour response rate was seen when the dopamine agonist cabergoline was added to docetaxel versus treatment with docetaxel alone (31/34 versus 13/36, *P *< 0.05), suggesting that endocrine prolactin protects against chemotherapy-induced cell death [12]. This corroborates preclinical data in breast and ovarian cancers and myeloma in which exogenous prolactin reduced the apoptotic response to commonly used cytotoxic agents [13-15]. Furthermore, breast cancer cell lines engineered to overexpress autocrine prolactin are resistant to taxane-mediated cell death both *in vitro *and in tumour xenografts *in vivo *[16].

Expression of prolactin has been demonstrated by *in situ *hybridisation and immunostaining in the epithelial component of over 90% of human breast cancer samples. Moreover, multiple breast cancer cell lines have been shown to synthesise and secrete bioactive prolactin *in vitro *[17,18]. Coexpression of the PRLR in breast cancer cells suggests that such prolactin may act in an autocrine/paracrine fashion to influence cell growth and survival. Approaches to antagonise autocrine prolactin in breast cancer cell lines have centred on prolactin-neutralising antibodies and PRLR antagonists. Neutralising prolactin antibodies have been shown to inhibit MCF-7 and T47Dco cell growth by 20% to 85% and to increase cell death twofold to threefold; however, no *in vivo *data have been reported on this approach [15,17]. In the latter study, prolactin neutralisation also resulted in additive augmentation of the apoptotic effects of exogenous ceramide [15].

The human PRLR antagonist G129R-hPRL was developed by site-directed mutagenesis, with the substitution of glycine for arginine at position 129 in the third alpha helix [19]. This mutation sterically hinders the sequential dimerisation and subsequent activation of the PRLR, but notably reduces the PRLR binding affinity 10-fold. G129R-hPRL has been shown to reduce Bcl2 expression and to induce apoptosis in both oestrogen receptor-positive and oestrogen receptor-negative breast cancer cell lines by one group, but this was not replicated by others [20,21]. Administration of G129R-hPRL reduced the growth of MCF-7 and T47D xenografts by 40% in immunocompromised mice compared with control mice, and its transgenic expression reduced the incidence of DMBA-induced mammary tumours by 50% [22].

The single G129R mutation does not remove all agonist activity, however, as demonstrated by high sensitivity assays *in vitro *and in the prostate gland *in vivo*, where G129R-hPRL was overexpressed using the metallothionein promoter [23]. These transgenic animals exhibited hypertrophied prostate glands with increased levels of activated mitogen-activated protein kinase compared with those from wildtype mice. Further modification of G129R by its N-terminal truncation results in a 'pure' PRLR antagonist, Δ1–9 G129R-hPRL (Δ1–9), which is devoid of any agonist activity. Coinjection of female Balb-c/J mice with Δ1–9 and exogenous prolactin reduced the prolactin-induced activation of STAT3 and STAT5 but only at an antagonist/prolactin ratio of 100:1. This confirms the antagonistic properties of Δ1–9 but also highlights the reduced binding affinity for the PRLR [23]. No further data are published on Δ1–9 in breast cancer, however it has been shown to induce apoptosis in prostate cancer cell lines by antagonising autocrine prolactin-mediated janus kinase 2 (JAK2)/STAT5A/B signalling [24].

In the current study we examined the effects of the 'pure' prolactin receptor antagonist Δ1–9 in breast cancer models *in vitro*. Although ineffective at inhibiting breast cancer cell line growth in monolayer culture as a single agent, Δ1–9 significantly augmented the cytotoxic effects of doxorubicin and paclitaxel. Short-term treatment with doxorubicin increased prolactin mRNA expression in all five cell lines tested. In addition, we demonstrate for the first time that prolactin receptor antagonism markedly inhibited the colony forming efficiency of cell lines and primary cancers *in vitro*. These data suggest a role for autocrine prolactin signalling in a subpopulation of clonogenic and treatment-resistant breast cancer cells that may be responsible for relapse after chemotherapy.

## Materials and methods

### Materials

Recombinant prolactin and Δ1–9 were produced in *Escherichia coli *as previously described and were gifts from V. Goffin, Necker, Paris, France [23]. MCF-7 and T47D (estrogen receptor alpha-positive) breast cancer cell lines and MDA-MB-453, MDA-MB-468 and SK-BR-3 (estrogen receptor alpha-negative) breast cancer cell lines were obtained from the European Collection of Cell Cultures (Porton Down, Salisbury, UK), and were maintained in DMEM (Invitrogen, Paisley, UK) supplemented with 10% FCS, 2 mM L-glutamine and 100 U/ml penicillin and streptomycin. Experimental media was phenol red free and contained 10% dextran-coated charcoal stripped serum unless otherwise indicated.

Cell lines were cultured in experimental medium for 48 hours before the initiation of experiments. Doxorubicin was diluted in normal saline and paclitaxel was diluted in dimethylsulphoxide. Equal concentrations of the diluents were used in controls, to a maximum dimethylsulphoxide concentration of 0.01% (at a paclitaxel dose of 1 μM), which had no effect on cell number compared with the experimental medium alone (data not shown).

### Sulphorhodamine B assay of cell number

The effects of Δ1–9 and cytotoxic agents on the cell number in monolayer culture were assessed using the sulphorhodamine B assay as described previously [25]. In brief, cells were seeded in 96-well plates, allowed to adhere for 24 hours and were treated with the desired experimental media, which was refreshed every 2 to 3 days. At harvest, monolayers were fixed with 10% trichloroacetic acid, washed and stained with 0.4% sulphorhodamine B in 1% acetic acid. Sulphorhodamine B was solubilised with 10 mM Tris base and the optical density read at 540 nm (Molecular Devices, Sunnyvale, CA, USA). Correlation coefficients for optical density versus cell number plated at 24 hours were >0.995 for all cell lines.

### Assessment of clonogenic growth in soft agar

A sandwich technique was used in which 1.5 ml of 1% high-melting-point Seakem GTG agar in PBS was poured into each well of a six-well plate and allowed to set for 10 to 20 minutes at room temperature. Breast cancer cell suspensions were drawn through a 24-gauge needle (0.45 mm) five to ten times to generate single-cell suspensions, before counting and re-suspension in agar in experimental media to achieve a final agar concentration of 0.3%. Two millilitres of the cell/agar mixture was added to each base layer and allowed to set for 10 to 20 minutes at room temperature before incubation at 37°C. Colonies >100 μm were counted after 14 to 21 days using an inverter microscope (Olympus UK Ltd, Watford Hertfordshire, UK). The colony-forming efficiency (CFE) was calculated as number of colonies/number of cells plated (%).

### Patients, ductal carcinoma *in situ *tissue and mammosphere assay

Ethical approval for the use of primary ductal carcinoma *in situ *(DCIS) samples was obtained from the South Manchester Local Regional Ethics Committee. Patients undergoing mastectomy following a diagnosis of DCIS gave fully informed consent for a sample of tumour to be excised and used in this research.

Excised samples were processed as previously described with minor modifications [26]. Samples were transferred immediately, dissected into 3-mm to 5-mm cubes and digested for 16 to 18 hours at 37°C in serum-free DMEM containing 200 U/ml type I collagenase (Worthington Biochemical Corporation, Lakewood, NJ, USA). The enzymatically digested tissue was then filtered sequentially through sterile 100-μm and 53-μm nylon meshes to obtain a single-cell suspension. The cells were then washed three times in DMEM:F12 medium and were resuspended in mammosphere culture medium comprising DMEM:F12 with the serum replacement supplement B27 (Invitrogen, Paisley, UK) and hydrocortisone, insulin, epidermal growth factor and bovine pituitary extract (SingleQuots; Cambrex Bio Science, Nottingham, UK). Cells were plated at a density of 500 cells/cm^2 ^into the wells of multiwell plates that had been coated with poly(2-hydroxyethyl methacrylate) to prevent adhesion. Colonies >60 μm were counted after 3 days of culture.

### Detection of prolactin mRNA by RT-PCR

RNA was extracted from breast cancer monolayers using TRIzol™ reagent according to the manufacturer's instructions (Invitrogen) and was reverse transcribed using the MMLVRT enzyme. Intron spanning oligonucleotide primers were designed for prolactin (forward, 5'-TGCCAGGTGACCCTTCGAGACCTG-3'; and reverse, 5'-GACTATCAGCTCCATGCCCTCTAG-3') and for the housekeeping gene acidic ribosomal phosphoprotein P0 (forward, 5'-TGGAAGTCCAACTACTTCCT-3'; and reverse, 5'-GAGAAGACCTCCTTTTTCCA-3').

PCRs were performed with Jump-start™ Red-Taq™ DNA polymerase (Sigma, Poole, UK). The PCR products were run on 2% agarose gels containing 0.5 μg/ml ethidium bromide and were visualised under UV illumination.

### Western analysis of proteins extracted from cell lysates and conditioned media

Monolayer cultures were placed on ice and the cells were washed with ice-cold stopping buffer (100 mM sodium orthovanadate in PBS) before being lysed with buffer containing 50 mM Tris–HCl, 150 mM NaCl, 1% Nonidet P40, 2 mM ethylenediamine tetraacetic acid, 1 mM sodium orthovanadate, 1 mM phenylmethylsulfonylfluoride, 10 mM NaF and a Complete™ mini protease inhibitor tablet (Roche Diagnostics Ltd, Burgess Hill, West Sussex, UK). Cells were scraped and suspensions placed on ice for 15 to 20 minutes. Cell debris was pelleted by centrifugation at 14,000 rpm for 15 minutes at 4°C. Protein concentrations of the supernatant were determined using the Bio-Rad protein assay according to the manufacturers' instructions (Bio-Rad Laboratories, Hercules, CA, USA), and lysates were boiled for 5 minutes in 'lane marker 5× reducing sample buffer' according to the manufacturer's instructions (Pierce Biotechnology, Cramlington, Northumberland, UK). The 5× buffer was used to facilitate loading of greater concentrations of protein lysate and volumes of conditioned media for electrophoresis.

For conditioned media experiments, approximately 3 million cells were cultured in 5 ml experimental medium per flask. Cells were treated with doxorubicin 1 μM for 8 or 24 hours before the experimental medium was removed and the cells washed in fresh medium containing 10% charcoal stripped serum. A further 5 ml medium was then added prior to harvest 40 or 24 hours later (that is, 48 hours from the start of the experiment). The conditioned media samples were centrifuged at 14,000 rpm for 15 minutes at 4°C to remove contaminants and were boiled in sample buffer at a 4:1 ratio for 5 minutes.

Conditioned media (40 μl) (and 10 μl sample buffer) or the required concentration of protein were electrophoresed on 10% to 12% polyacrylamide gels according to the method of Laemmli [27], and were transferred to nitrocellulose membranes. Nonspecific binding was blocked with 5% fat-free milk in Tris-buffered saline containing 0.1% Tween-20, before overnight incubation at 4°C with the primary prolactin antibody (1:100 clone 127813; R&D Systems, Abingdon, UK) diluted in 3% fat-free milk in Tris-buffered saline containing 0.1% Tween-20. Blots were washed three times in Tris-buffered saline containing 0.1% Tween-20 before goat anti-mouse secondary antibody (1:1000; Autogen Bioclear UK Ltd, Calne, Wiltshire, UK) incubation for 1 hour at room temperature. Immunoblots were developed using Supersignal West Pico chemiluminescent substrate (Pierce Biotechnology) according to the manufacturer's instructions, and images captured on a Chemi-8000 cooled image digital camera (UVP Ltd, Cambridge, UK).

### Dual-label fluorescence immunocytochemistry

Cell lines were seeded into the wells of eight-well chamber slides at a density of 50,000 cells/well and were allowed to adhere for 48 hours. Cells were fixed in 4% formalin in PBS for 15 minutes and cell membranes were permeablised with 0.1% Triton X-100 in PBS. Nonspecific binding sites were blocked with 10% goat serum in PBS containing 1% BSA. The prolactin antibody (clone 127813; R&D Systems) was labelled with green fluorescent Alexa Fluor according to the manufacturer's instructions (Zenon Mouse IgG Labelling kit; Molecular Probes, Paisley, UK).

Unlabelled PRLR primary antibody (clone B6.2; Neomarkers, Fremont, CA, USA) was applied to the cells first at a concentration of 1:100 for 1 hour at room temperature followed by a Texas red conjugated goat anti-mouse secondary antibody (1:1000; Autogen Bioclear UK Ltd, Calne, Wiltshire, UK) for 1 hour at room temperature in the dark. The Alexa Fluor-labelled prolactin antibody was then added (1:100) for 30 minutes at room temperature in the dark. Negative controls consisted of native and Alexa Fluor-labelled isotype-specific mouse IgG incubated under identical conditions.

Slides were mounted using Vectorshield fluorescent mounting fluid containing 4',6-diamidino-2-phenylindole to counterstain cell nuclei. Stained cell monolayers were examined using a Zeiss Axioscope microscope and the images were captured using a Nikon camera and Meta Vue software (Universal Imaging, Downington, PA, USA). Exposure times for each filter (red, green and blue) were standardised across all cell lines and negative controls.

## Results and discussion

**Figure 1:**
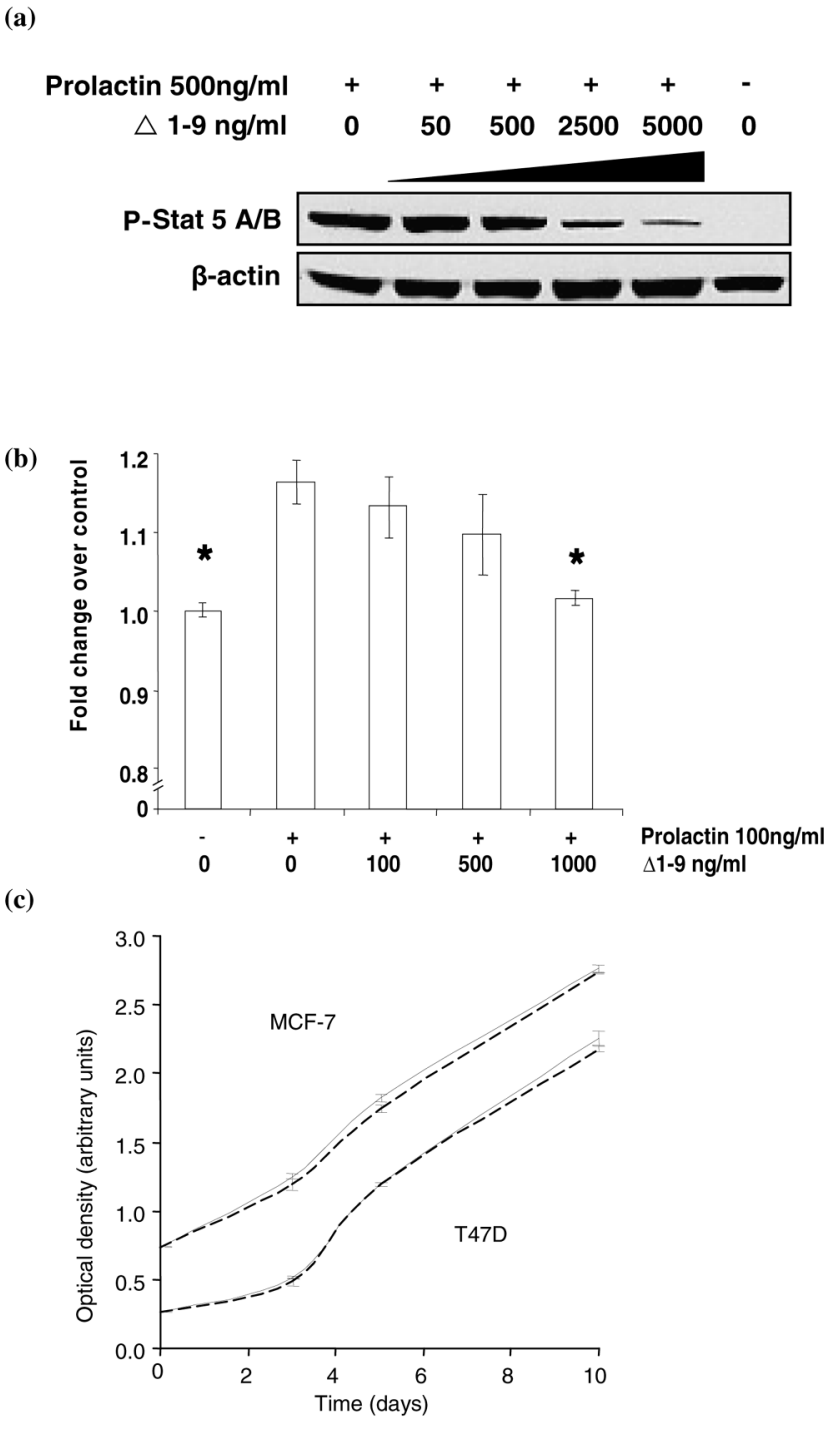
Δ1–9-G129R-hPrl inhibits exogenous prolactin effects but does not reduce cell numbers in standard growth conditions. **(a) **Western analysis of T47D cell lysates confirms prolactin (500 ng/ml for 30 min) induced phosphorylation of STAT 5A/B, which is abrogated in a dose-dependent manner by coincubation with Δ1–9-G129R-hPrl (Δ1–9). **(b) **Prolactin-induced increase in cell number of T47D cells in monolayer culture for 3 days was abrogated by coincubation with Δ1–9. **(c) **1,000 ng/ml Δ1–9 (broken lines) had no effect on cell number in control medium (solid lines) of MCF-7 cells (upper two curves) or T47D cells (lower two curves) in a monolayer culture in the absence of exogenous prolactin. *P < 0.05 by Student *t *test compared with 100 ng/ml prolactin in the absence of Δ1–9. Error bars, standard error of the mean of triplicate observations. All data representative of at least three experiments.

The capacity of Δ1–9 to antagonise the effects of exogenous prolactin on activation of its downstream signalling intermediates and growth was confirmed in T47D cells (Figure 1a,b). At the exogenous prolactin concentrations employed, a 10-fold excess of Δ1–9 was required for almost complete antagonism of prolactin-induced stimulation. This necessity is consistent with data demonstrating a 10-fold reduced binding affinity of site 2 mutated lactogenic hormones for their cognate receptors, compared with their native ligands [23]. In the development of the growth hormone receptor antagonist Peg-Visomant (Somavert™), this need was overcome by further engineering the site 1 binding site, with eight additional amino acid substitutions, restoring the receptor binding affinity to that of native growth hormone [28]. A similar approach to increase the binding affinity of the PRLR antagonists to the PRLR would be required if they are to be developed successfully as therapeutic agents.

In the absence of exogenous human prolactin, Δ1–9 had no effect on the T47D and MCF-7 cell numbers in monolayer culture over 10 days (Figure 1c). At Δ1–9 concentrations between 1 and 10,000 ng/ml in 2-day to 10-day assays in media containing either FCS or charcoal stripped serum, no significant effect on the cell number in a monolayer of any of the five breast cancer cell lines tested (MCF-7, T47D, MDA-MB-453, MDA-MB-468 and SKBR3) could be demonstrated (data not shown). The lack of activity of this PRLR antagonist in monolayer culture is consistent with previously published work [21]. Using similar culture conditions to ours, no effects on cancer cell proliferation or apoptosis were seen with the parental PRLR antagonist G129R. In contrast, using the same antagonist, Chen and colleagues demonstrated small but statistically significant reductions in cell number in T47D and MCF-7 cell lines correlated to an increase in apoptosis [20]. These latter data are consistent with reports of growth inhibition of breast cancer cell lines using prolactin inhibitory antibodies, and it is possible that subtle variations in culture conditions/constituents or genetic drift of the cell lines may account for the discrepancies between our findings and those of others [15,17,29].

**Figure 2:**
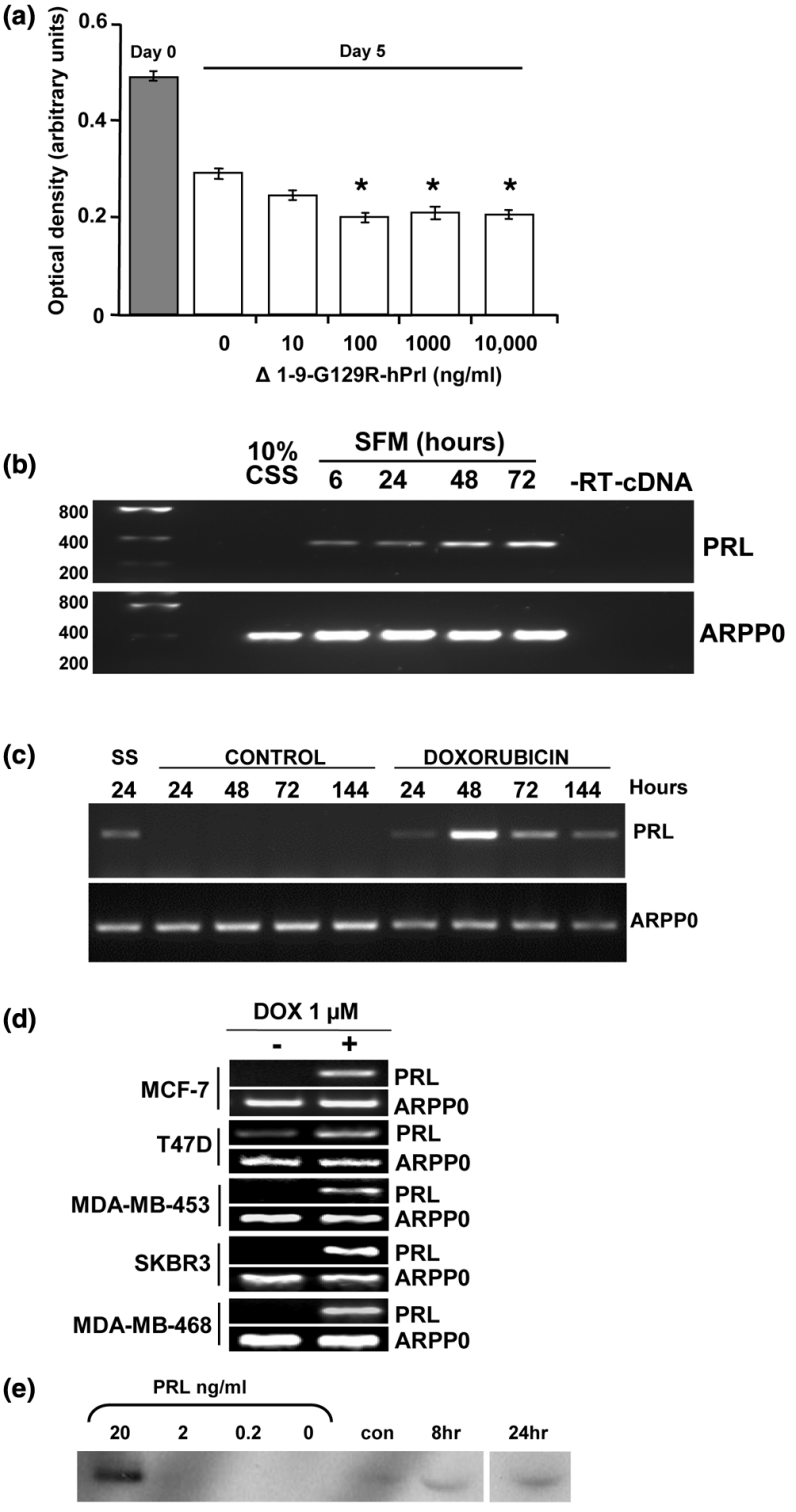
Autocrine prolactin production is stimulated by serum starvation and treatment with doxorubicin. **(a) **Monolayer culture of MCF-7 cells in serum-free medium over 5 days (open bars) resulted in cell loss compared with the cell number at 24 hours after plating in 10% charcoal stripped serum (CSS) (grey bar). Δ1–9-G129R-hPrl (Δ1–9) increased cell loss in a dose-dependent manner (clear bars). Prolactin (PRL) mRNA was detected in MCF-7 cells by RT-PCR in response to **(b) **serum starvation and **(c) **treatment with 1 μM doxorubicin for 24 hours, but not in cells grown in control medium (10% CSS). ARPP0, acidic ribosomal phosphoprotein P0; SFM, serum free medium. Following doxorubicin treatment for 24 hours, cultures were washed and fresh control medium was added. Times correspond to the number of hours from the start of the experiment. **(d) **All five cell lines were treated with doxorubicin (DOX) as in (c) and were harvested at 48 hours, and they demonstrate the induction of prolactin mRNA expression. ARPP0 was used as a housekeeping gene loading control in all RT-PCR experiments. **(e) **Western analysis of prolactin in cell culture media. Bracketed lanes represent the positive control concentration curve of recombinant human prolactin. Other lanes are conditioned media from MCF-7 cells in 10% CSS alone (con) or following 1 μM doxorubicin treatment for 8 and 24 hours and conditioned media harvested 48 hours after the start of the experiment.

In serum-free medium, the MCF-7 cell number declined over 5 days in culture and, in contrast to the findings above, Δ1–9 induced further significant reductions in the MCF-7 cell number of 5% to 10% over controls, at concentrations >10 ng/ml (Figure 2a). Using RT-PCR, a time-dependent increase in prolactin mRNA expression was seen in response to serum starvation, leading to the hypothesis that autocrine prolactin is produced to promote survival of breast cancer cells under conditions of stress, and that its antagonism reduces cell survival (Figure 2b). To examine the effects of more therapeutically relevant stressors, MCF-7 cells were exposed to 1 μM doxorubicin for 24 hours before RNA extraction. Doxorubicin treatment was associated with an increase in prolactin mRNA expression, which was sustained for at least 5 days after treatment, whereas it could not be detected in cells cultured in control medium (Figure 2c).

All five of the breast cancer cell lines tested demonstrated increased prolactin mRNA expression after a 24-hour exposure to 1 μM doxorubicin compared with untreated cells (Figure 2d). The increases in prolactin mRNA were accompanied by increases in prolactin protein secretion, as demonstrated by western analysis of the conditioned medium from these cells (Figure 2e). Prolactin was secreted into the medium at concentrations between 2 and 20 ng/ml following doxorubicin treatment, with increased secretion evident with treatment for 24 hours. As approximately 3 million cells were used in 5 ml medium (see Materials and methods) and the amount of prolactin produced by the cells is taken as approximately 5 ng/ml, this secretion equates to ~25 ng/3 × 10^6 ^cells/24 hours, which is equivalent to ~8 pg/cell/24 hours. This level is comparable with the results of Ginsburg and Vonderhaar, who detected 7 to 14 pg/cell/24 hours in T47Dco cells as assessed by the Nb2 bioassay using concentrated conditioned media [17]. No prolactin was detected in the cell lysates prepared from these experiments (data not shown), consistent with previous observations that prolactin is not stored in intracellular granules in extrapituitary tissues, in contrast to the pituitary gland [30].

**Figure 3:**
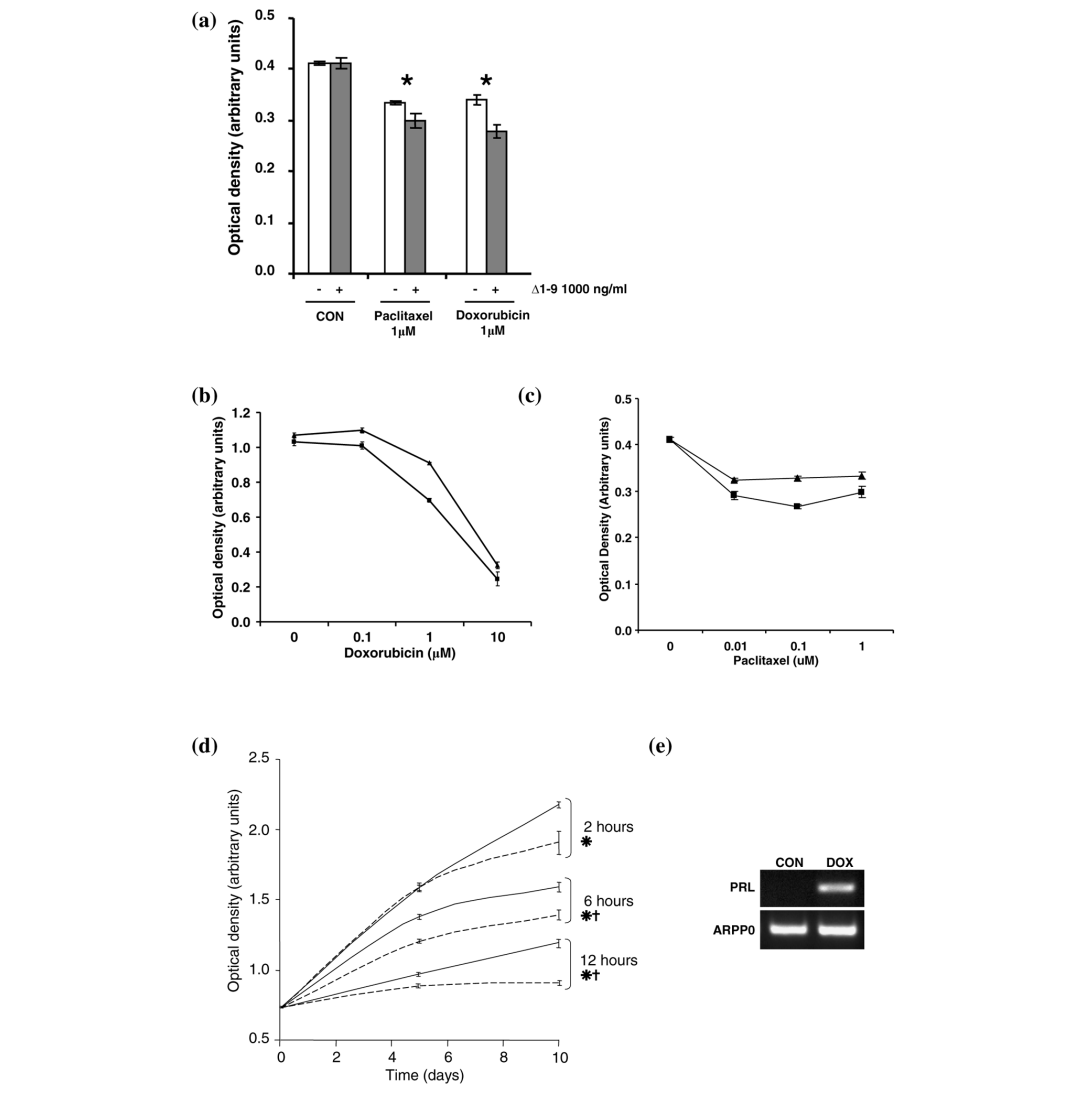
Δ1–9-G129R-hPrl reduces the MCF-7 cell number in combination with doxorubicin or paclitaxel. **(a) **Sulphorhodamine B assay of MCF-7 cells treated for 48 hours with chemotherapy or control medium (10% charcoal stripped serum (CON)) alone (empty bars) or in combination with 1,000 ng/ml Δ1–9-G129R-hPrl (Δ1–9) (filled bars). *P < 0.05 by Student *t *test between paired bars. Forty-eight-hour MCF-7 dose–response curves to **(b) **doxorubicin, and **(c) **paclitaxel alone (▲) or in combination with 1,000 ng/ml Δ1–9 (■). **(d) **MCF-7 cells in monolayer culture treated with 1 μM doxorubicin for pulse durations of 2, 6 and 12 hours before removal of doxorubicin and subsequent culture in control medium in the absence (solid lines) or presence (broken lines) of 1,000 ng/ml Δ1–9. Medium was refreshed every 2 to 3 days thereafter. *P *< 0.05 by Student *t *test at 5 days (*) and 10 days (†). **(e) **MCF-7 cells treated with 1 μM doxorubicin (DOX) for 24 hours were cultured until exponential growth resumed (5 to 6 weeks) and were analysed for prolactin (PRL) mRNA expression. ARPP0, acidic ribosomal phosphoprotein P0.

We next examined whether Δ1–9 potentiated the effects of doxorubicin, and of the microtubule stabilising agent paclitaxel, on cell number. Combining doxorubicin or paclitaxel at a concentration of 1 μM with 1,000 ng/ml Δ1–9 produced small reductions in cell number compared with chemotherapy alone, suggesting that autocrine prolactin produced under these conditions functions as a survival factor (Figure 3a). This effect was seen over a range of chemotherapy doses in short-term culture (Figure 3b,c) and also in longer term culture of cells for 10 days, after short initial pulses of doxorubicin for 2 to 12 hours (Figure 3d). Coculture with Δ1–9 significantly reduced the cell number by 10% to 15% compared with cells grown in control medium, irrespective of the initial doxorubicin pulse duration. Cells that survived treatment with 1 μM doxorubicin, to repopulate the culture dish, expressed increased levels of prolactin mRNA compared with cells not exposed to the drug (Figure 3e).

Previous *in vitro *studies have demonstrated additive effects of antiprolactin approaches with ceramide or cytotoxic agent-induced cell death in breast cancer cell lines [15,20]. In addition, the engineered overexpression of prolactin in the MDA-MB-468 breast cancer cell line protected the cells from paclitaxel-induced apoptosis [16]. Similar observations have also been made in ovarian cancer and prostate cancer as well as in the plasma cell malignancy myeloma, suggesting that prolactin may alter the sensitivity to cytotoxic agents in a wide variety of malignancies [13,14,31].

The data presented here are the first to demonstrate that the production of biologically relevant autocrine prolactin is induced by chemotherapeutic agents. Interestingly, the phenomenon of increased prolactin production in response to anthracyclines has been observed in rats *in vivo*, albeit from lactotrophs in the anterior pituitary gland [32]. Such chemotherapy-induced systemic prolactin release may help to explain the beneficial effects of cabergoline when coadministered with chemotherapy in women with metastatic breast cancer, in the clinical trial cited above [12].

**Figure 4:**
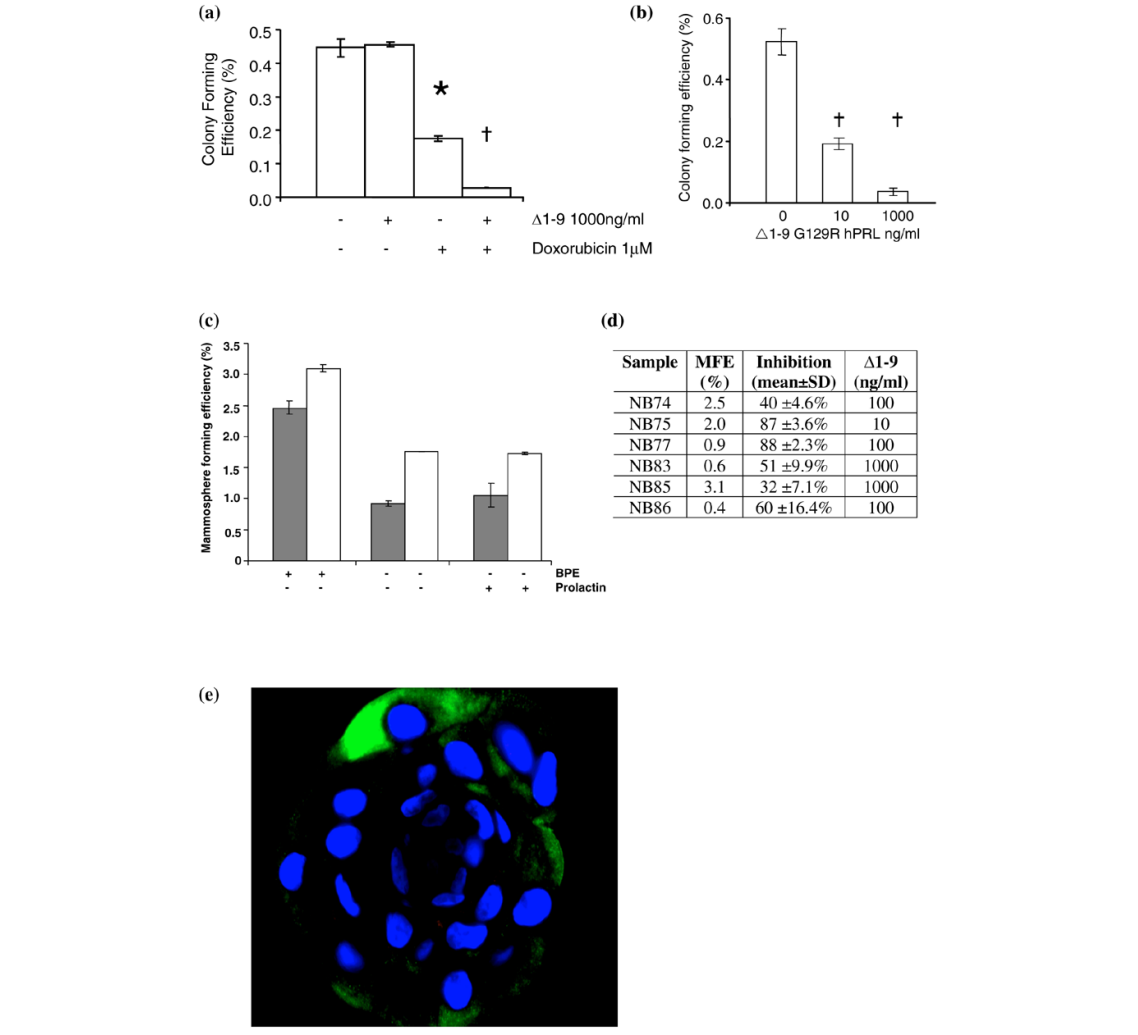
Single-agent Δ1–9-G129R-hPrl inhibits colony formation of cell lines and primary ductal carcinoma *in situ *samples. **(a) **MCF-7 cells treated for 2 hours in monolayer culture with control medium, Δ1–9-G129R-hPrl (Δ1–9) (1,000 ng/ml), doxorubicin (1 μM) or the combination were subsequently plated into soft agar (see Materials and methods) and colonies were counted at 14 days. * p < 0.05 compared to untreated control and Δ1–9 alone, † p < 0.01 compared to each of the other three conditions (both by Student t-test). **(b) **MCF-7 cells plated into soft agar with Δ1–9 incorporated into the agar at the concentrations shown. **(c) **Effects of bovine pituitary extract (BPE) and prolactin on the ductal carcinoma *in situ *(DCIS) mammosphere forming efficiency. DCIS mammospheres were counted on day 3 in complete culture medium (complete medium), complete medium without BPE (-BPE) and with 500 ng/ml human prolactin substituted for BPE (-BPE + PRL). Grey-filled and empty bars represent the means of duplicate observations from two independent samples; error bars, standard error of the mean. **(d) **Table of the six DCIS samples detailing their mammosphere forming efficiency (MFE), the maximum reduction in MFE with Δ1–9 and the concentration of Δ1–9 at which maximum inhibition was achieved. **(e) **Photomicrograph of a DCIS mammosphere following embedding in paraffin and immunohistochemical analysis for the PRLR using a Fluorescein isothiocyanate (FITC)-labelled secondary antibody (green fluorescence). Cell nuclei counterstained using 4',6-diamidino-2-phenylindole (blue). Scale bar = 50 μm.

The soft agar assay was used to investigate the effects of Δ1–9 on the clonogenic growth of breast cancer cell lines both alone and after treatment with doxorubicin. Treatment of MCF-7 cells for 2 hours with 1 μM doxorubicin in monolayer culture, before plating them into soft agar, reduced the CFE by 61% (*P *< 0.05 by unpaired Student *t *test; Figure 4a). The addition of 1,000 ng/ml Δ1–9 to the doxorubicin further reduced the CFE, resulting in a 94% reduction compared with controls (*P *< 0.01) and an 84% reduction compared with doxorubicin treatment alone (*P *< 0.01). In the absence of doxorubicin, Δ1–9 pretreatment had no effect on MCF-7 CFE in soft agar (Figure 4a).

In contrast, incorporation of Δ1–9 into both layers of the agar resulted in a marked, dose-dependent reduction in the MCF-7 CFE (Figure 4b). A maximal reduction in the CFE of 90 ± 4.2% (mean ± standard deviation; *P *< 0.001) was seen at 1,000 ng/ml Δ1–9 after 14 days of culture. The MDA-MB-453 and SKBR3 cells did not form colonies in this assay. The T47D and MDA-MB-468 cell lines did from colonies, however, with a similar CFE as MCF-7 cells. Δ1–9 reduced their CFEs by 89 ± 3.8% and 93 ± 6.8%, respectively, at a concentration of 1,000 ng/ml (*P *< 0.001 for both). This dramatic effect of PRLR antagonism in clonogenic cells but not in a monolayer culture suggests the presence of a subpopulation of clonogenic cells with increased dependence upon autocrine/paracrine prolactin signalling compared with their nonclonogenic counterparts.

**Figure 5:**
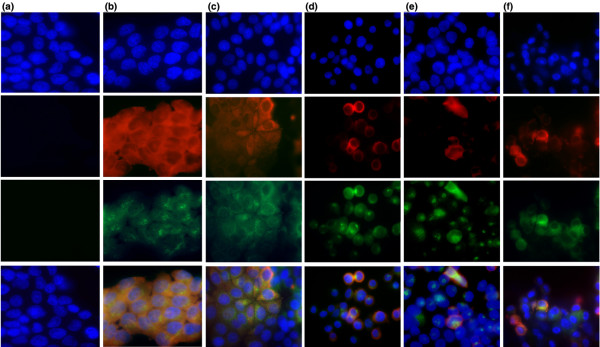
Immunofluorescence of prolactin and the prolactin receptor in breast cancer cell lines. Dual-fluorescence immunocytochemistry for the prolactin receptor (PRLR) and prolactin in breast cancer cell lines. Cells were plated onto eight-well chamber slides at identical cell densities and were allowed to adhere for 48 hours prior to fixation and staining (see Materials and methods). Top row, 4',6-diamidino-2-phenylindole; second row, PRLR–Texas red; third row, prolactin–Fluorescein isothiocyanate (FITC) (green); fourth row, merged images. **(a) **Negative controls photographed with identical exposure and processed with identical software settings, compared with antibody-positive samples shown in subsequent columns: **(b) **T47D cells, **(c) **MCF-7 cells, **(d) **MDA-MB-468 cells, **(e) **MDA-MB-453 cells, and **(f) **SKBR3 cells.

The expression of both the PRLR and autocrine prolactin was thus examined in a monolayer culture of breast cancer cell lines by dual-label fluorescence immunocytochemistry. All T47D cells showed strong staining for both the PRLR (red fluorescence) and for prolactin (green fluorescence), which showed subcellular localisation in a region consistent with Golgi localisation (Figure 5b). Such subcellular localisation may explain why prolactin could be detected by immunocytochemistry, due to the increased concentrations at these sites, but not by western analysis of whole cell lysates. Western analysis of subcellular fractions may facilitate detection of prolactin from cell lysates. The staining pattern of MCF-7 cells for the PRLR was more heterogeneous and was confined to particular colonies of cells. These cells also coexpressed prolactin and were often surrounded by cells that expressed neither prolactin nor its receptor (Figure 5c). The three oestrogen receptor-negative cell lines displayed less frequent PRLR-positive cells compared with the oestrogen receptor-positive lines (Figure 5d to 5f). Further work is required to correlate the relative clonogenicity of cells with expression levels of PRLR and prolactin, and would be augmented by improvements in PRLR antibodies to determine the relative expression levels of the long form of the PRLR and the dominant negative short form of the PRLR.

Only one other group has reported data on clonogenic assays and prolactin in breast cancer cells; in their hands, prolactin increased the CFE by up to 25% [2]. To our knowledge, the present study is the first to demonstrate that an antiprolactin approach can significantly reduce the colony-forming ability of breast cancer cells in culture. Next the effects of Δ1–9 on the clonogenicity of disaggregated primary DCIS specimens were examined. These DCIS cells did not grow in the soft agar assay described, so a suspension culture technique termed the mammosphere assay was employed. This was chosen on the basis that a highly tumorigenic subpopulation of primary breast cancer cells with the properties of both self-renewal and multipotency can be isolated by means of their capacity to survive anoikis and to grow in suspension culture [33].

Mammospheres were generated in suspension culture from six DCIS samples. Such mammospheres have been shown to be of epithelial origin, as they express cytokeratins 14 and 18, but were shown to form faster and with greater efficiency than those from normal breast tissue – confirming their origin from the malignant cells rather than adjacent normal breast [26]. As bovine pituitary extract was used in the mammosphere growth medium, it was necessary to first investigate the effects of the bovine prolactin content on DCIS mammosphere formation. In the first two mammosphere assays on DCIS samples, therefore, controls were set up in which 500 ng/ml exogenous human prolactin was substituted for the bovine pituitary extract. The results demonstrate that the removal of bovine pituitary extract reduced mammosphere formation significantly but that human prolactin could not substitute for the effects of bovine pituitary extract on mammosphere formation (Figure 4c). In view of these and previously published data demonstrating the reduced affinity of bovine prolactin for the human PRLR, we were satisfied that exogenous prolactin from bovine pituitary extract would not interfere significantly with the effects of Δ1–9 on autocrine prolactin signalling [17].

In all six DCIS samples tested, the mammosphere forming efficiency was reduced significantly by the inclusion of Δ1–9 into the culture medium – with maximal inhibition of mammosphere forming efficiency at concentrations from 10 to 1,000 ng/ml (Figure 4d). An example of inhibition is shown in Figure 4d. The median reduction in the mammosphere forming efficiency at 3 days was 56% (range 32% to 88%). Immunohistochemistry of paraffin-embedded DCIS mammospheres demonstrated cytoplasmic expression of the PRLR in cells located at their periphery (Figure 4e).

We have demonstrated a disparity between the effects of Δ1–9 in routine growth in monolayers and under conditions of cell stress or clonogenic growth. In response to cell stressors such as doxorubicin, signalling components known to be downstream of the PRLR, notably Src and focal adhesion kinase, are upregulated in cells lines [34,35]. The balance between cell apoptosis and proliferation downstream of PRLR signalling has been described previously to be dependent on the relative abundance of such signalling intermediates [1,36-38]. Changes in the abundance of signalling intermediates in this situation could therefore induce autocrine/paracrine prolactin dependence and preferential survival of prolactin-expressing cells. Furthermore, cells exhibiting such dependence may be a subpopulation of clonogenic cells, antagonism of which in a short-term monolayer culture results in no discernable change in cell number. Conditions of cell stress in monolayer and clonogenic assays expose the relative importance of these cell populations, the growth of which can be antagonised by Δ1–9.

The mechanisms by which autocrine prolactin secretion protects breast cancer cell lines from the effects of serum starvation and cytotoxic agents have not yet been elucidated. Other groups have shown that overexpression of prolactin in breast cancer cell lines induces the expression of the antiapoptotic protein Bcl-2, and that treatment with G129R-hPrl has been shown to reduce Bcl-2 levels whilst increasing those of the proapoptotic protein Bax [4,39]. The inability of a second group to repeat these findings may be due to the effect being evident only in a subpopulation of clonogenic cells and not their more differentiated progeny. Differences in cell culture techniques, reagents and cell lines between laboratories may have exacerbated these discrepancies, and these differences highlight the need for better techniques to identify such treatment-resistant subpopulations of cells.

The expression and secretion of autocrine/paracrine prolactin mRNA and protein following chemotherapy raises the possibility of improved breast cancer therapy with the combination of cytotoxic chemotherapy and a PRLR-targeted agent. The increase in mRNA expression to a maximum at 48 hours, followed by a fall in levels thereafter, suggests that combination therapy should be given initially. The duration of PRLR-targeted therapy would probably be at least 1 week as mRNA was detectable up to this point. Investigation of longer term prolactin mRNA expression post treatment was not investigated, however, and the most efficacious treatment regimens will need to be discerned from further *in vitro *studies and xenograft studies *in vivo*.

## Conclusion

In contrast to its lack of effect in monolayer culture, PRLR antagonism with Δ1–9 profoundly inhibits colony formation of both breast cancer cell lines and primary tumour samples. This suggests the presence of a clonogenic population of breast cancer cells that are preferentially sensitive to prolactin inhibition. In addition, autocrine/paracrine prolactin appears to be a survival factor in this subpopulation of breast cancer cells and is induced by treatment with doxorubicin or paclitaxel, as treatment with the PRLR antagonist Δ1–9 potentiates the efficacy of such drugs. If autocrine prolactin is preferentially produced by a cytotoxic-resistant putative breast cancer stem cell, then the rational combination of cytotoxic agents and Δ1–9 may improve outcomes in breast cancer therapy.

## Abbreviations

Δ1–9 = Δ1–9-G129R-hPrl; BSA = bovine serum albumin; CFE = colony-forming efficiency; DCIS = ductal carcinoma *in situ*; DMEM = Dulbecco's modified Eagle's medium; FCS = foetal calf serum; PBS = phosphate-buffered saline; PCR = polymerase chain reaction; PRLR = prolactin receptor; RT = reverse transcriptase; STAT = signal transducer and activator of transcription.

## Competing interests

The authors declare that they have no competing interests.

## Authors' contributions

SJH designed and performed the experiments, analysed the data and wrote the manuscript. GF performed the DCIS mammosphere experiments. TH performed the western analyses. EA and RBC participated in the study design and writing of the manuscript.
